# Supplementary material for: Splice-Junction-Based Mapping of Alternative Isoforms in the Human Proteome
Source: Cell Rep. Author manuscript; Available in PMC 2020 Jan 15. (PMC6961840; doi:10.1016/j.celrep.2019.11.026)
Supplement: 2 [file NIHMS1546469-supplement-2.zip › DF1/PXD009737/TestisTrypsin/TestisTrypsin_29_MMS19_DQVAAGPLAAPMGALWGLVHDFVVGQQEGPADQVAADVK.pdf]

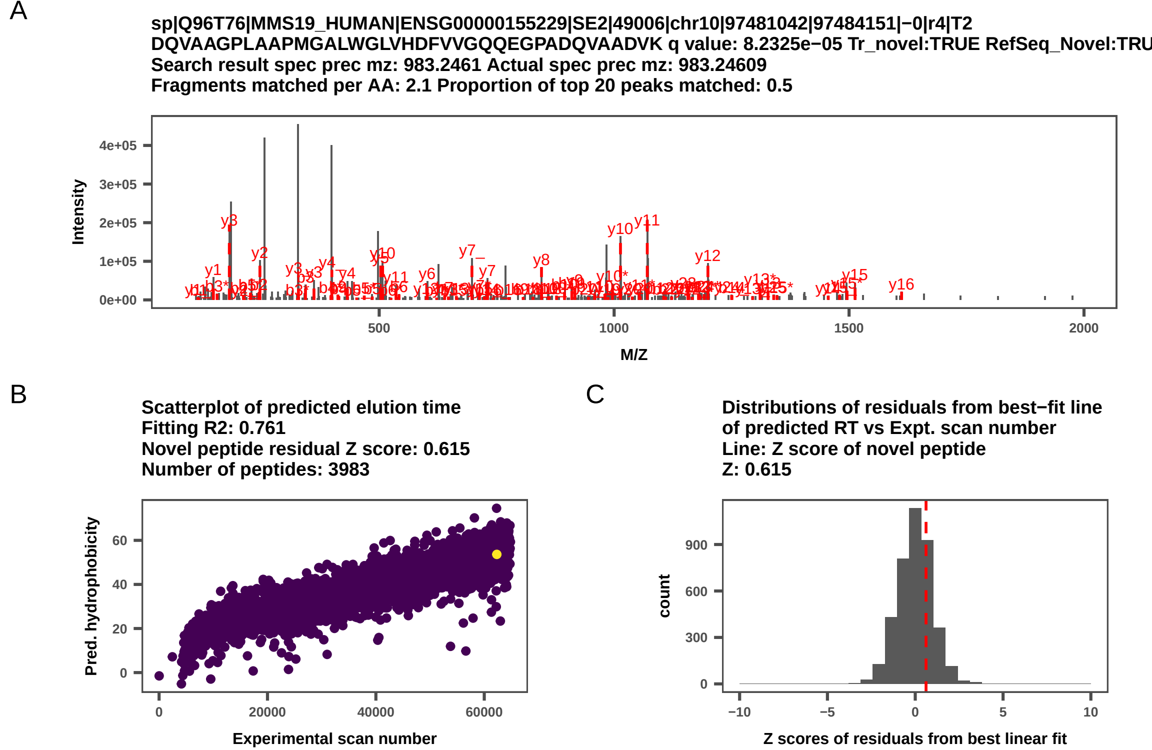
A
sp|Q96T76|MMS19_HUMAN|ENSG00000155229|SE2|49006|chr10|97481042|97484151|−0|r4|T2
DQVAAGPLAAPMGALWGLVHDFVVGQQEGPADQVAADVK q value: 8.2325e−05 Tr_novel:TRUE RefSeq_Novel:TRU
Search result spec prec mz: 983.2461 Actual spec prec mz: 983.24609
Fragments matched per AA: 2.1 Proportion of top 20 peaks matched: 0.5
Intensity
4e+05
3e+05
2e+05
1e+05
0e+00
M/Z
500
1000
1500
2000
y3
y2
y1
y4
y6
y7
y8
y10
y11
y12
y15
y16
B
Scatterplot of predicted elution time
Fitting R2: 0.761
Novel peptide residual Z score: 0.615
Number of peptides: 3983
Pred. hydrophobicity
60
40
20
0
Experimental scan number
0
20000
40000
60000
C
Distributions of residuals from best−fit line
of predicted RT vs Expt. scan number
Line: Z score of novel peptide
Z: 0.615
count
900
600
300
0
Z scores of residuals from best linear fit
−10
−5
0
5
10
